# Spin crossover-induced colossal positive and negative thermal expansion in a nanoporous coordination framework material

**DOI:** 10.1038/s41467-017-00776-1

**Published:** 2017-10-20

**Authors:** Benjamin R. Mullaney, Laurence Goux-Capes, David J. Price, Guillaume Chastanet, Jean-François Létard, Cameron J. Kepert

**Affiliations:** 1 School of Chemistry, The University of Sydney, Building F11, Sydney, NSW 2006 Australia; 2 ICMCB, UPR CNRS 9048, Université Bordeaux I, 87 Av. du Doc. A., Schweitzer, F-33608 Pessac France

## Abstract

External control over the mechanical function of materials is paramount in the development of nanoscale machines. Yet, exploiting changes in atomic behaviour to produce controlled scalable motion is a formidable challenge. Here, we present an ultra-flexible coordination framework material in which a cooperative electronic transition induces an extreme abrupt change in the crystal lattice conformation. This arises due to a change in the preferred coordination character of Fe(II) sites at different spin states, generating scissor-type flexing of the crystal lattice. Diluting the framework with transition-inactive Ni(II) sites disrupts long-range communication of spin state through the lattice, producing a more gradual transition and continuous lattice movement, thus generating colossal positive and negative linear thermal expansion behaviour, with coefficients of thermal expansion an order of magnitude greater than previously reported. This study has wider implications in the development of advanced responsive structures, demonstrating electronic control over mechanical motion.

## Abstract (web summary)

Controlling mechanical motions in solid state devices is highly desirable for the development of nanoscale machines. Here, Kepert and colleagues exploit an ultra-flexible coordination framework in which thermally-controlled Fe(II) spin transitions result in remarkable flexing of the crystal lattice.

## Introduction

Control of mechanical function in solid state devices requires precise manipulation of material structure. Such functional components commonly employ vibrational or electronic mechanisms, such as magnetostriction^1^ and the piezoelectric effect^2^ which may be controlled, for example, through thermal, electrical and magnetic stimuli. However, the achievement of prominent push–pull mechanical action in a material presents a major challenge due to the large magnitude of controlled structural change required. One approach is through stimuli-responsive organic assemblies, such as electroactive polymers^3^, and positionally switchable polymeric rotaxanes^4^, which have potential as artificial molecular muscles. An alternative strategy is to target geometrically flexible crystalline materials which can undergo induced conformational change.

Materials that exhibit anomalous thermal expansion properties are deeply important to understand and strategically design thermomechanical behaviour^5^. It is well understood that most materials exhibit positive thermal expansion (PTE) as higher temperatures increase the amplitude of atomic bond vibrations^6^, for which the relative rate of thermal expansion^7^, *α*, usually lies within the range 0 × 10^−6^ K^−1^ < α < 20 × 10^−6^ K^−1^. Near-zero thermal expansion or negative thermal expansion (NTE) can arise from a range of different mechanisms, including phase transitions such as magnetostriction in ferromagnetic materials^8^, valence transitions in intermetallic^9^ and fulleride^10^ materials, and the population of low-energy phonon modes, such as is observed in flexible oxide- or cyanide-bridged framework materials^11, 12^. Colossal thermal expansion^13^, in which the coefficient of thermal expansion has a magnitude, |α| > 100 × 10^−6^ K^−1^, is of intense interest for generating the structural change necessary for thermomechanical action, and has been observed to arise through vibrational^13, 14^, and intermetallic charge transfer^15^ mechanisms.

Here we exploit the electronic phenomenon of spin crossover, which is very strongly coupled to the crystal lattice, to achieve unprecedented thermal mechanical function, as observed through extreme PTE and NTE. The spin crossover phenomenon, in which a metal ion switches between different electronic spin states, is a reversible transition that can be induced by multiple external inputs, such as temperature, pressure, guest or light irradiation^16–19^. The strong electron–lattice coupling is due to changes in the geometry and strength of coordination bonding at the metal site. Exploiting this structure–property relationship, recent reports have shown that spin crossover materials can be used to create light-induced molecular actuators^20, 21^. Herein we present the ultra-flexible framework, [Fe(bpac)(Au(CN)_2_)_2_]·2EtOH (bpac = 1,2-bis(4′-pyridyl)acetylene), noted hereafter as [Fe], in which an electronic transition affects the delicate interplay of weak interactions in the framework, producing a dramatic mechanical effect on the lattice. Furthermore, strategically diluting the framework with Ni(II) disrupts the cooperativity of the spin crossover, resulting in continuous colossal thermal expansion over the transition temperature range.

## Results

### Framework lattice structure

**Fig. 1:**
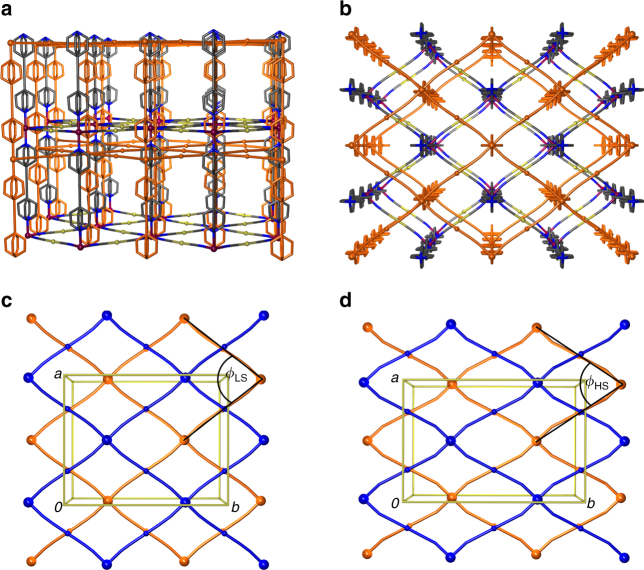
Single crystal structure of [Fe]. **a** at 190 K viewed along the *a*-axis, and **b**, the *c*-axis directions. The lattice conformation of the {Fe(Au(CN)_2_)_2_} sheets in the *ab*-plane is shown for comparison at 190 K **c**, and 240 K **d**. Note the relative LS–HS change in the unit cell dimensions: Δ*a* = −6.8%; Δ*b* = + 10.2%. Au···Fe···Au angles: *ϕ*_LS_ = 76.85°; *ϕ*_HS_ = 67.77°. Fe and Au centres are represented by larger and smaller spheres respectively and the interpenetrating nets are shaded *blue* and *orange*. Hydrogen atoms and disordered solvent are omitted for clarity

The single-crystal X-ray structure of [Fe] at 190 K is shown in Fig. 1a, b. The framework consists of rhombic grids of {Fe(Au(CN)_2_)_2_} which lie parallel to the *ab*-plane and are pillared perpendicularly by disordered bpac ligands, forming a three-dimensional net. The length of the Au(CN)_2_^−^ and bpac ligands results in sufficient pore space for a second identical net to be interpenetrated within the first. The relative orientation of these nets is driven by strong aurophilic interactions, as evidenced by a characteristic Au···Au distance between the adjacent metal cyanide grids of 3.0843(5) Å. Modelling of the electron density in the pore space revealed 2 ethanol molecules per formula unit.

Single crystal structures obtained at 190 and 240 K reveal the framework to be in the low spin (LS) and high spin (HS) states respectively, as identified by the mean Fe–N bond length (1.96(4) Å at 190 K; 2.17(9) Å at 240 K)^22^. As expected, the crystallographic *c* parameter increases from LS to HS, and correlates well with the increase in the Fe–N bond lengths. However, the change in the *a* and *b* parameters is so pronounced that it cannot be attributed to the change in bond length alone: from the LS to HS state, the parametric changes are Δ*a* = −0.850(3) Å and Δ*b* =  + 1.610(5) Å, which are observed crystallographically as an increased lattice compression (Fig. 1c, d).

### Lattice flexing mechanism

To understand the mechanism for this remarkable behaviour, it is necessary to consider the various structural energetics involved. First, there is expected to be very little energy penalty associated with scissor-type motion of the rhombic {Fe(Au(CN)_2_)_2_} grids, with the framework topology being highly underconstrained, allowing weak intermolecular interactions to affect the grid geometry. Factors that influence this include weak inter-network ligand–ligand interactions and possible host–guest interactions, which appear to favour distortion of the framework away from a regular orthogonal geometry, leading to bond characteristics such as non-linear Au–CN–Fe linkages and a distorted Fe(N)_6_ octahedral coordination. At temperatures above the transition, this framework distortion is geometrically allowed by the weaker bonding of the HS Fe(II) centres, which can adopt a distorted octahedral geometry with non-linear coordination of the cyanide groups. At 240 K the Fe(II) octahedral distortion parameter (the sum of the deviations from 90° for all *cis* bond angles), Σ_HS_ = 19°, and the acute Au···Fe···Au angle,* θ*_HS_ = 67.77°. Conversely, LS Fe(II) is energetically driven to become more regularly octahedral, with a more linear coordination of the cyanide ligands, due to the stronger Fe–N bonds, and optimisation of metal–ligand orbital interactions of this spin state. These changes in the lattice conformation result in {Fe(Au(CN)_2_)_2_} rhombic grids with a structure that is closer to an orthogonal conformation. At 190 K the Fe(II) Σ_LS_ is reduced to 10° and the acute Au···Fe···Au angle,* θ*_LS_ increases to 76.85°. It is clear that the scissor-like lattice flexing behaviour is partially driven by the Fe(II) coordination geometry, as the more rigidly orthogonal LS environment induces a lattice conformation such that the acute Au···Fe···Au angle is also closer to 90°, resulting in expansion along the *a*-axis, and contraction along the *b*-axis.

This lattice flexing behaviour has not been previously observed in spin crossover materials, with the predominant behaviour being anisotropic PTE through the spin transition. The ‘Hofmann-type’ framework series incorporates tetracyanidometallate (*M* = Ni, Pd, Pt) components, which are rigidly restrained to a near-orthogonal conformation of the metal cyanide grids^23, 24^, while the structurally analogous [Fe(bipytz)(Au(CN)_2_)_2_] (bipytz = 3,6-bis(4-pyridyl)-1,2,4,5-tetrazine) framework conformation is driven by strong inter-lattice interactions which energetically preclude any significant flexing of the framework^25^.

### Spin crossover behaviour of [Fe]

**Fig. 2:**
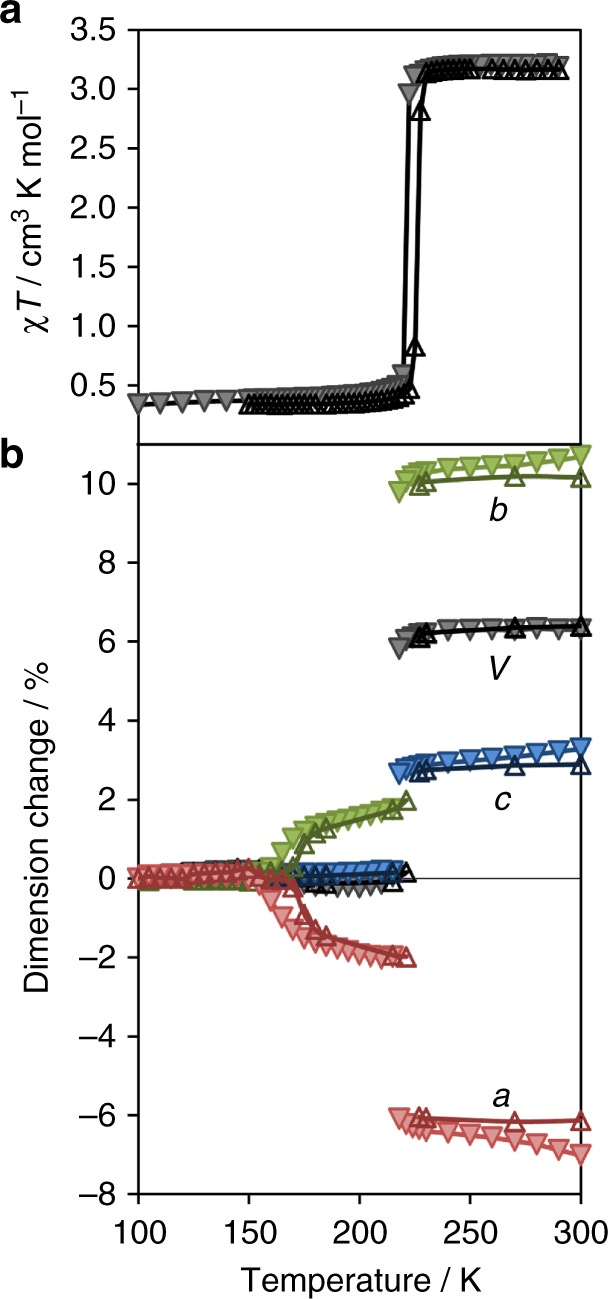
Variable temperature behaviour of [Fe]. **a** Molar magnetic susceptibility product *χ*_M_ *T vs*. temperature. **b** the percentage change in lattice dimension for *a, b, c* and volume. Data are presented upon (*filled triangle*) cooling and (*unfilled triangle*) warming

The spin transition properties of [Fe] were studied using variable temperature magnetic susceptibility (Fig. 2a). The material undergoes an abrupt spin transition, with a cooling HS (^5^T_2g_) to LS (^1^A_1g_) transition temperature of $T_{1/2}^ \downarrow$=221 K and a heating LS to HS transition temperature of $T_{1/2}^ \uparrow$=226 K, producing a hysteresis width of ~ 5 K. Differential scanning calorimetry (DSC) data for this material are consistent with the presence of this reversible spin transition, showing an exothermic peak at 220 K on cooling, and an endothermic peak at 228 K on warming (Supplementary Fig. 13).

To study the structural behaviour of the framework above and below the spin transition, variable temperature synchrotron powder X-ray diffraction was employed, and modelled using Le Bail fits to yield lattice parameters^26^. As shown in Fig. 2b, the spin transition results in phase discontinuities at ~ 215 and ~ 221 K for the cooling and warming datasets respectively, over which there are large changes in the *a*, *b* and *c* parameters. From the LS (185 K) to HS (230 K) state, Δ*a* = −0.606(1) Å and Δ*b* =  + 1.343(2) Å. Below the spin transition the scissor-type lattice flexing continues until a second hysteretic transition occurs between 172 and 162 K, which is not electronic in origin (as is consistent with both the magnetic and DSC data, the latter indicating that only a small enthalpic change occurs; Supplementary Fig. 13a), producing additional lattice changes from 140 to 185 K of Δ*a* = −0.213(2) Å and Δ*b* =  + 0.203(2) Å. In total from 140 to 230 K, the *a* parameter contracts by 6.2% and the *b* parameter expands by 10%. Indeed, the degrees of structural change are comparable with a number of notable materials which exhibit large abrupt phase transitions, including ~ 7% in a rotor compound^27^ and ~ 8% in an organometallic martensite^28^. While the relative dimension changes for [Fe] are less than previously reported for certain porous materials^29, 30^, importantly they are achieved through an electronic mechanism, which can be manipulated to produce controlled, continuous motion through lattice modification.

### Metal dilution for continuous colossal thermal expansion

The observation of extreme transition-induced changes in the lattice structure prompted us to investigate methods to produce continuous lattice motion. Temperature-dependent rates of dimension change cannot be determined for a discontinuous phase transition, but disruption of lattice cooperativity to induce a gradual structural transition in the bulk material enables extraction of coefficients of thermal expansion. The sharpness of a spin transition is understood to occur predominantly through long-range elastic interactions between metals centres^31, 32^, and it has been shown that dilution with non-spin crossover metal sites disrupts the lattice cooperativity, resulting in more gradual spin transition behaviour^33^.

**Fig. 3:**
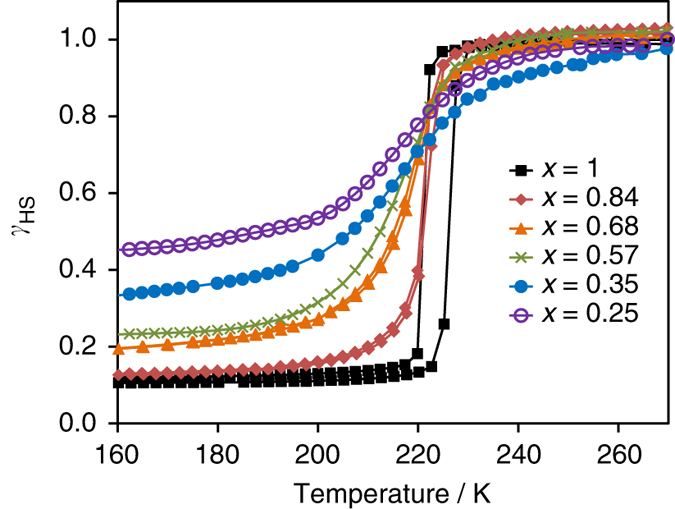
Effect of metal dilution on spin crossover. The Fe(II) high spin fraction (γ_HS_) was calculated from variable temperature magnetic susceptibility data for [Fe_*x*_Ni_1−*x*_]. *Lines* are included for visual clarity

To apply this strategy, we required a metal with a coordination volume that is intermediate between that of HS and LS Fe(II) so as to minimise the dopant effect on the spin transition temperature. For this reason, we chose to introduce Ni(II) dopant sites into the [Fe] framework. The resulting materials, [Fe_*x*_Ni_1**−***x*_(bpac)(Au(CN)_2_)_2_]·2EtOH (denoted hereafter as [Fe_*x*_Ni_1−*x*_]), are isostructural with the pure [Fe] material. Temperature-dependent magnetic susceptibility studies on [Fe_*x*_Ni_1−*x*_] demonstrated that Ni(II) dilution results in a more gradual spin transition, as anticipated, with a higher Ni(II) proportion resulting in a wider temperature range of the transition, and an increased residual HS fraction below the transition (Fig. 3).

**Fig. 4:**
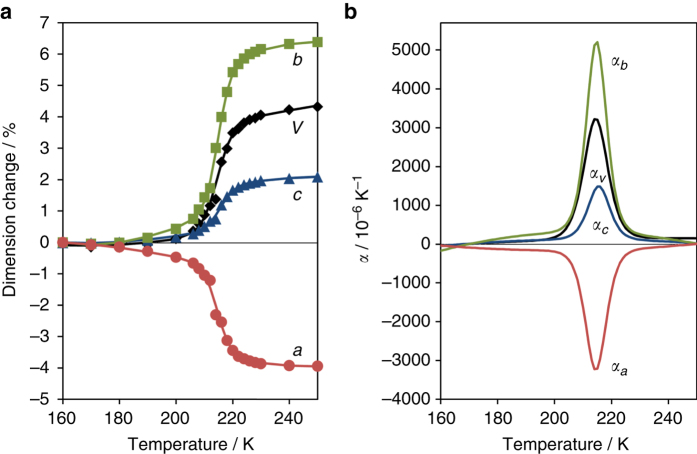
Temperature-dependence on the structure of [Fe_0.84_Ni_0.16_]. **a** Lattice parameters (*markers* represent data; *line* represents fitted model): *a, b, c* and volume. **b** Thermal expansion coefficients (from model), *α*: for *a, b, c* and volume

The gradual spin crossover behaviour of the [Fe_*x*_Ni_1−*x*_] series is directly coupled with continuous lattice motion over the spin transition, as observed by powder X-ray diffraction. Importantly, the diffraction data also indicate that this behaviour arises from the bulk homogeneous material, rather than from a distribution of varying dilution levels within different crystallites (Supplementary Fig. 5). Continuous thermomechanical motion is clearly demonstrated by the [Fe_0.84_Ni_0.16_] material, which exhibits extreme lattice flexing (Fig. 4a) that can be modelled to determine coefficients of thermal expansion (Fig. 4b; Supplementary Note 4; Supplementary Movie for mechanism animation). At 215 K, the linear coefficients of thermal expansion along the *a*, *b* and *c* axes are α_*a*_ = −3200 × 10^−6^ K^−1^, α_*b*_ =  + 5200 × 10^−6^ K^−1^ and α_*c*_ =  + 1500 × 10^−6^ K^−1^ respectively. To our knowledge, these continuous NTE and PTE values are an order of magnitude greater than any reported to date.

**Fig. 5:**
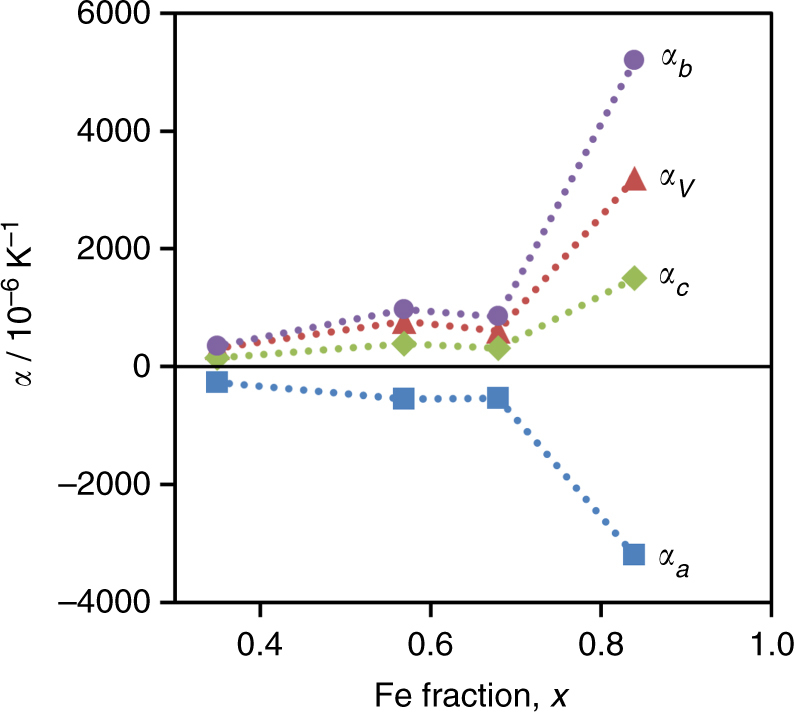
Maximum coefficients of thermal expansion for [Fe_*x*_Ni_1−*x*_]. These values were calculated using a model fit to the lattice parameter data. *Lines* are included as a visual guide

As increasing the Ni(II) dilution broadens the spin transition temperature, the colossal linear thermal expansion similarly occurs over a wider temperature range, with a concomitant reduction in its peak magnitude. As shown in Fig. 5, the maximum coefficients of thermal expansion decrease from *x* = 0.84 to 0.68, though a colossal magnitude is still observed, with |α| > 500 × 10^−6^ K^−1^ for the *a* and *b* parameters at 216 K. Further dilution to *x* = 0.57 does not significantly affect the spin crossover behaviour, but at *x* = 0.35 the transition is more gradual and incomplete, and the maximum thermal expansion coefficients decrease accordingly.

## Discussion

Controlling the energetics of spin switching has enabled access to an unprecedented magnitude of extreme linear thermal expansion behaviour. By coupling an electronic transition with an ultra-flexible crystal lattice, mechanical motion is generated by changes in the metal coordination behaviour. Here we have demonstrated that the weak energetic perturbation of temperature can generate extreme lattice movement and, moreover, that fine control of this effect can be engineered through variation in framework composition. It is also well established that spin transitions are accessible through other stimuli, such as pressure^34^, guest effects^18^ or light irradiation^35^. These could potentially allow access to new methods to induce mechanical change, perhaps paving the way toward light-activated artificial muscles.

## Methods

### Sample Preparation

Single crystals of framework [Fe] were grown by slow diffusion of a 1:2:1 molar ratio of Fe(ClO_4_)_2_·9H_2_O, K[Au(CN)_2_] and 1,2-bis(4′-pyridyl)acetylene (bpac) in ethanol. Bulk powder was synthesised by fast mixing of the same components, with substitution of varying molar ratios of Ni(ClO_4_)_2_·6H_2_O for the Ni(II)-diluted species, [Fe_*x*_Ni_1−*x*_]. The crystal structures of the bulk synthesis products were confirmed by powder X-ray diffraction, and sample purity by elemental analysis (see Supplementary Methods).

### Single-crystal X-ray diffraction

Reflection data were collected on a Bruker-Nonius FR591 Kappa APEX II equipped with Mo-Kα (0.71073 Å) and an Oxford Instruments nitrogen gas cryostream. The crystal was first quench-cooled in the cryostream at 100 K, then data collections were performed at 190 and 240 K, below and above the spin crossover transition, respectively. Both structures were solved in the orthorhombic space group *Cmma*. Disordered guest ethanol molecules were modelled inside the pores of both structures (2.0 EtOH per formula unit).

### Powder diffraction studies

Variable temperature powder X-ray diffraction experiments were conducted on the Powder Diffraction beamline at the Australian Synchrotron. Unit cell parameters were modelled with a Le Bail fit, using the GSAS^26^ and EXPGUI^36^ software packages. Thermal expansion parameters were calculated by fitting a model function to the variable temperature unit cell data. The model included a sigmoidal component to reflect the transition temperature range, and a polynomial component to describe the temperature range outside the transition (Supplementary Note 4).

### Data availability

Crystal structure data for the structures reported in this study have been deposited at the Cambridge Crystallographic Data Centre (CCDC). These data have been allocated deposition nos. CCDC 1501291, 1501292 and 1534111, and can be obtained free of charge from the CCDC (http://www.ccdc.cam.ac.uk/datarequest/cif).

Further data that support the findings of this study are available from the corresponding author upon reasonable request.

## Electronic supplementary material

Supplementary Information

Description of Additional Supplementary Files

Supplementary Movie 1
