# Supplementary material for: Spin crossover-induced colossal positive and negative thermal expansion in a nanoporous coordination framework material
Source: Nat Commun. 2017 Oct 20;8:1053. doi: 10.1038/s41467-017-00776-1 (PMC5648752; doi:10.1038/s41467-017-00776-1)
Supplement: Supplementary file 1 — Supplementary Information [file 41467_2017_776_MOESM1_ESM.pdf]

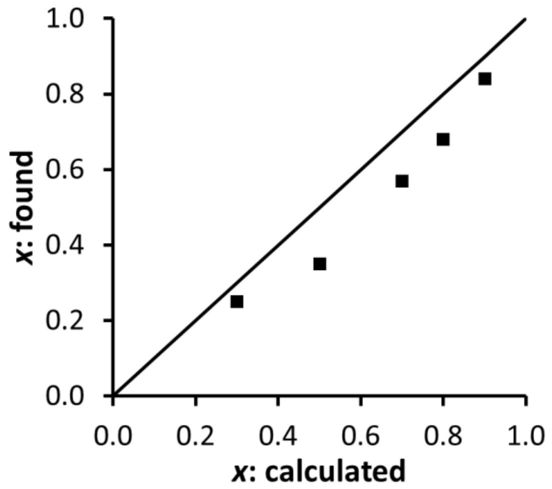

Supplementary Figure 1 | Plot of the iron fraction values for the **[Fe_xNi_{1-x}]** series as calculated from the relative molar amounts of metallic salts used in the syntheses, *versus* that found from elemental analysis. The divergence from the line indicates a slight preference for Ni over Fe in the framework crystallization.

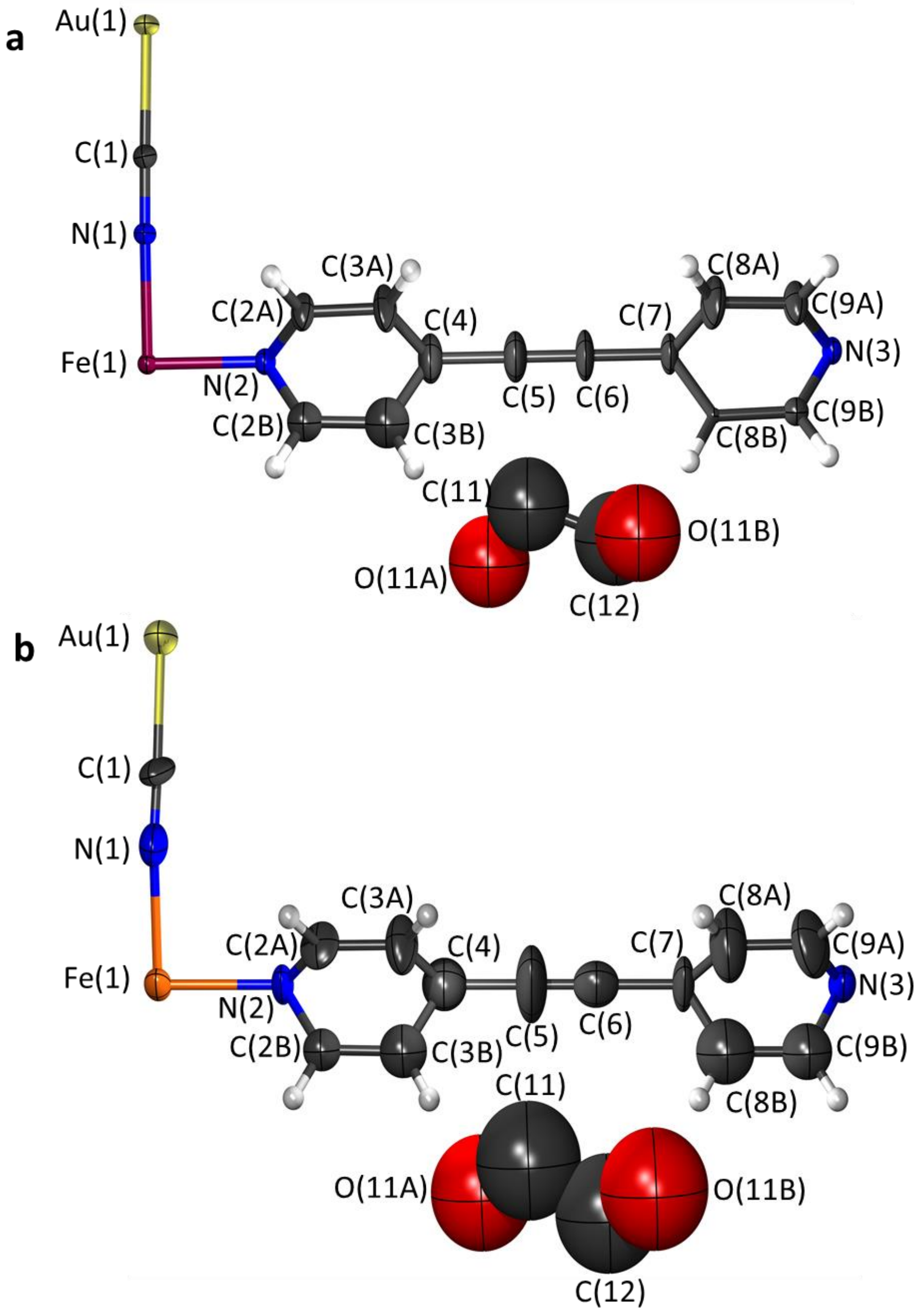

Supplementary Figure 2 | Asymmetric units of **[Fe]** at (**a**) 190 K, and (**b**) 240 K, showing thermal ellipsoids at 50% probability.

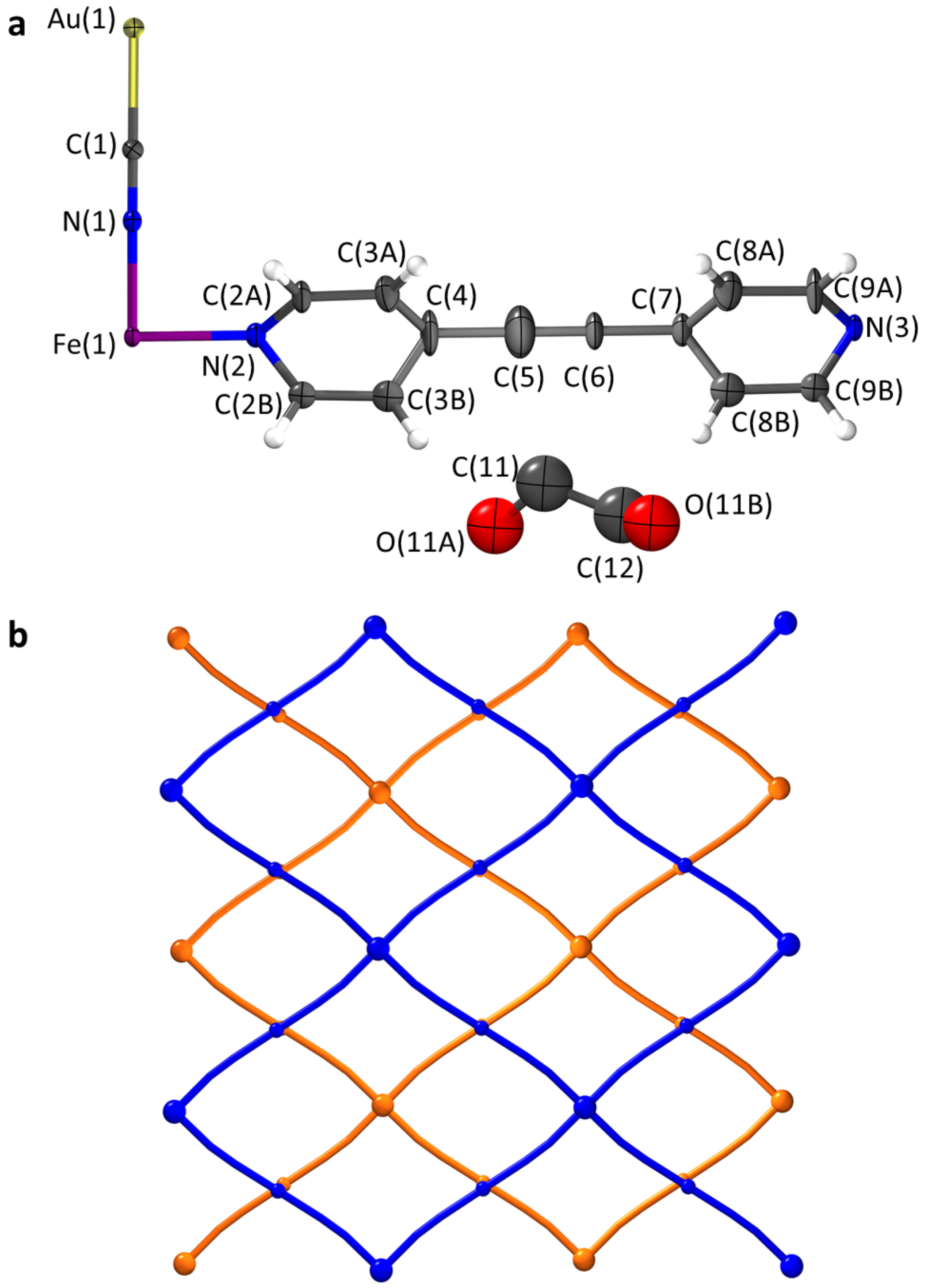

Supplementary Figure 3 | Single crystal structure of **[Fe]** at 100 K: **a**, asymmetric unit, showing thermal ellipsoids at 50% probability; and **b**, the metal cyanide grid as viewed along the *c*-axis direction, with Fe and Au atoms represented by large and small spheres respectively, and the interpenetrating nets shaded blue and orange.

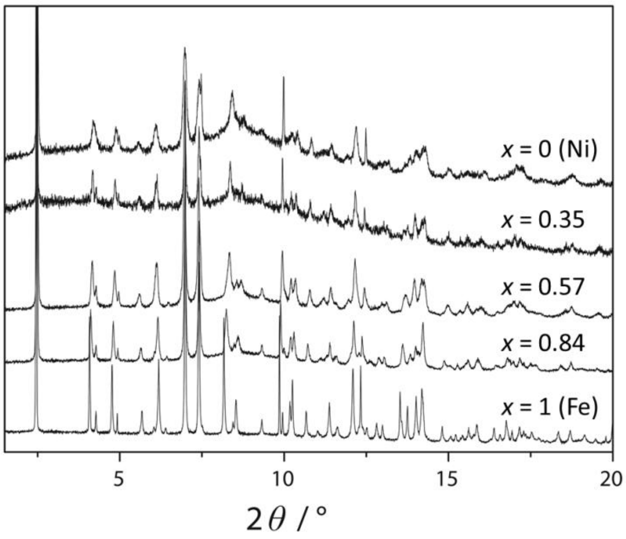

Supplementary Figure 4 | Synchrotron X-ray powder diffractograms for **[Fe_xNi_{1-x}]** at 250 K.

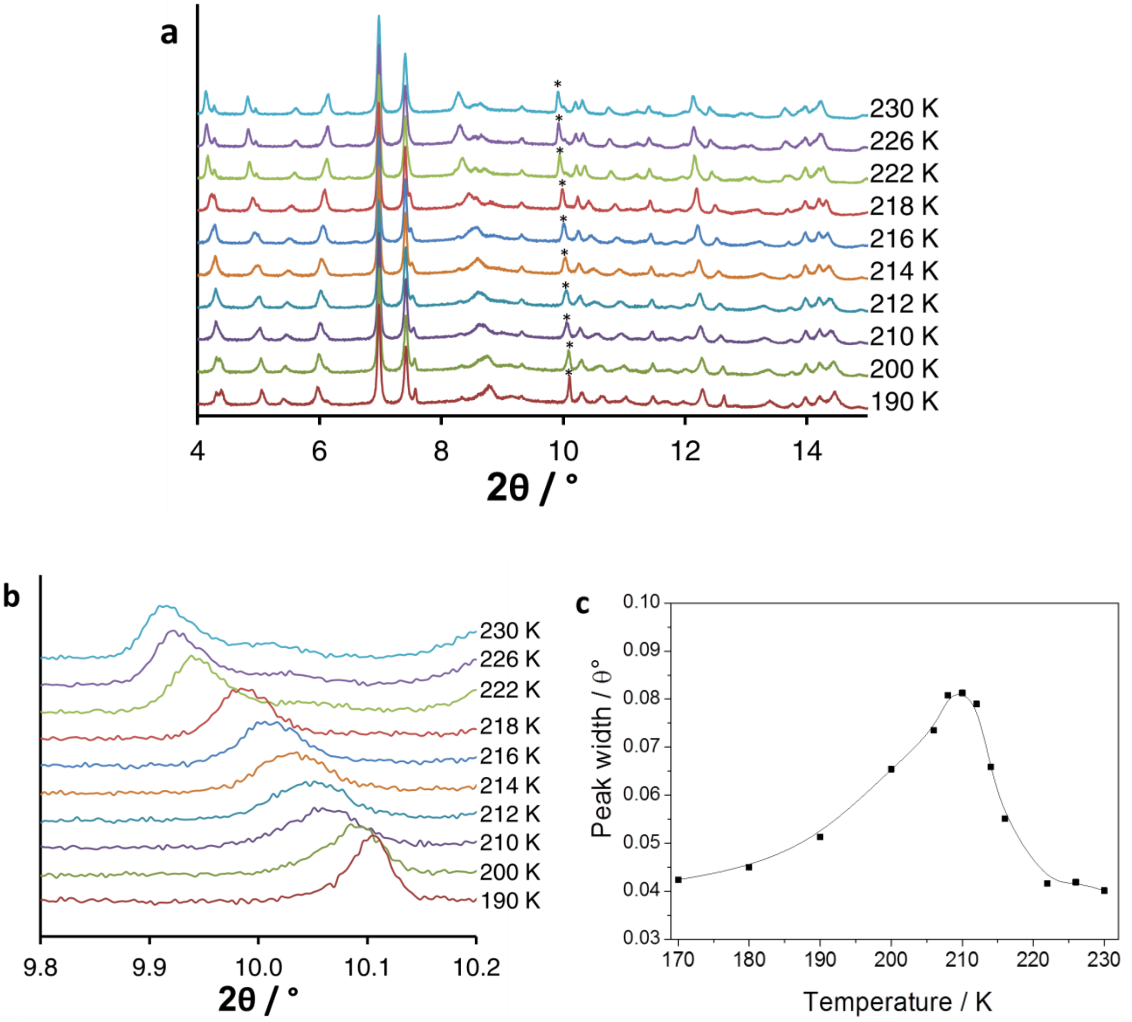

Supplementary Figure 5 | a, Variable temperature synchrotron powder X-ray diffraction patterns for ethanol-solvated **[$Fe_{0.84}Ni_{0.16}$]**, showing dramatic shifts in some peak positions (e.g.: (020) ~4.3→4.1°; (200) ~5.4→5.6°; and (004) ~10.1→9.9° [starred peak]), and near-invariance in others (e.g.: (220) ~7.0°; (221) ~7.4°). **b**, Movement of the (004) reflection over the spin transition. **c,** Peak width of the (004) reflection *versus* temperature. The peak width was fitted with lamp.exe[8] using a Pseudo-Voigts model. The line is included as a visual guide.

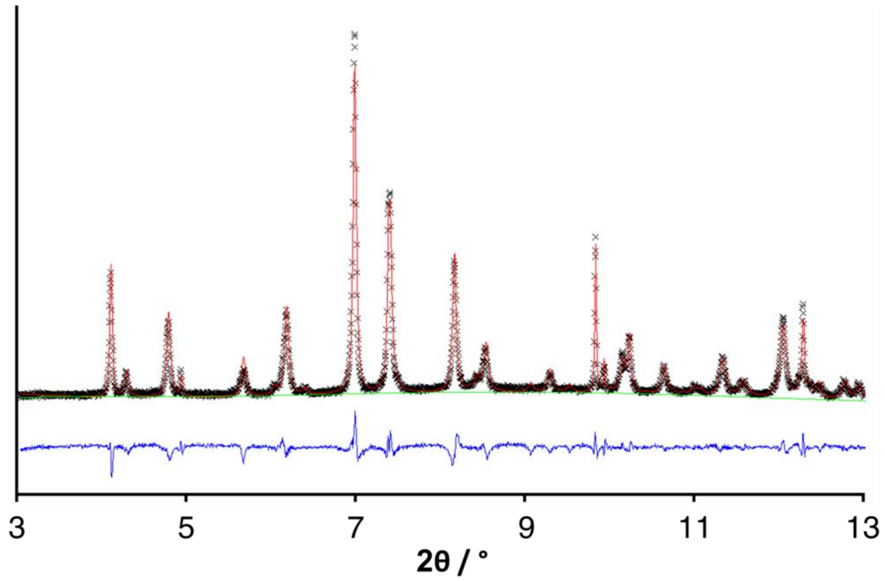

Supplementary Figure 6 | Le Bail fit to the synchrotron powder X-ray diffraction pattern of **[Fe]** at 300 K. Diffraction data points are represented as crosses (×), the model fit is the solid red line and the difference between the model and the data is displayed by the blue line.

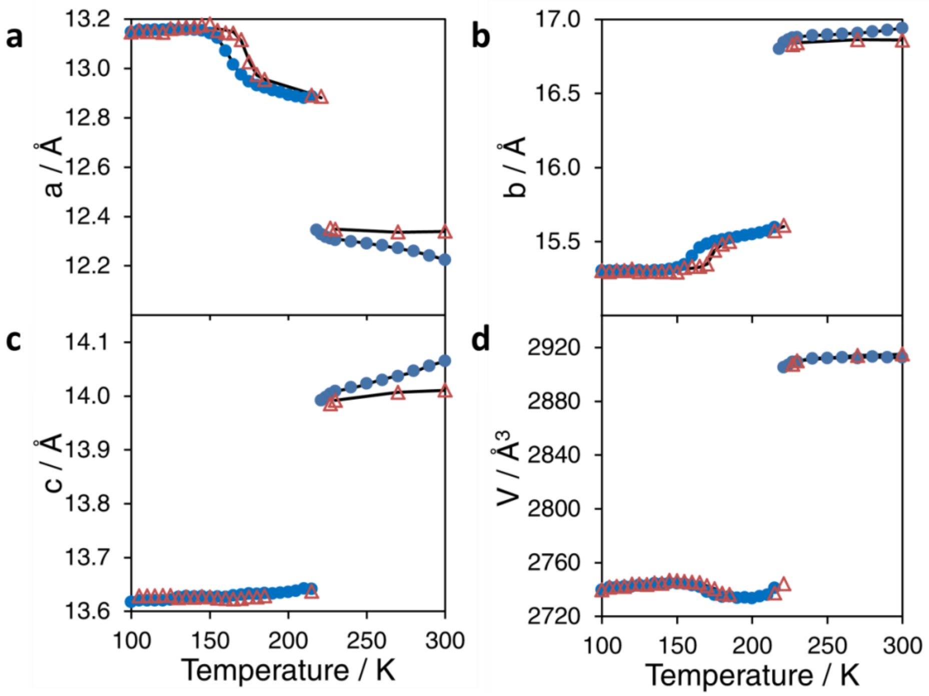

Supplementary Figure 7 | Unit cell dimensions (points) for **[Fe]** from 100 to 300 K, with supplementary equation 1 model fit (line): **a**, *a* parameter. **b**, *b* parameter. **c**, *c* parameter. **d**, volume. Data are presented upon ● cooling and △ warming.

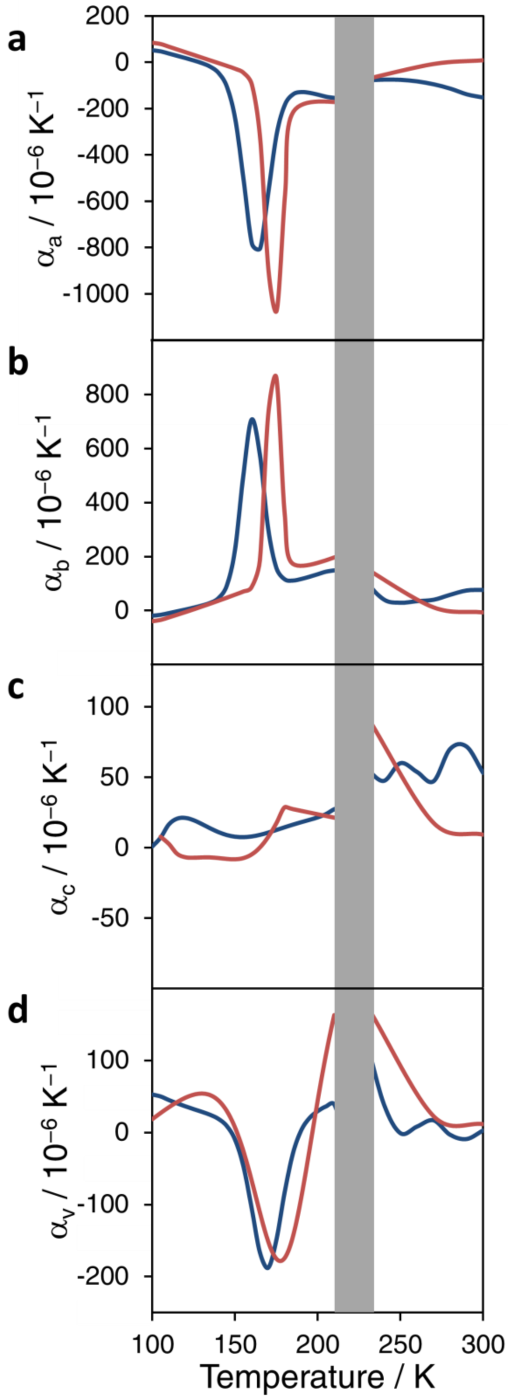

Supplementary Figure 8 | Thermal expansion coefficients of **[Fe]** from 300 to 100 K (blue), and 100 to 300 K (red) for: **a**, *a* parameter. **b**, *b* parameter. **c**, *c* parameter. **d**, volume. Grey bars denote the discontinuity associated with the spin transition. See Supplementary Fig. 6 for the corresponding plots of variable temperature lattice parameters.

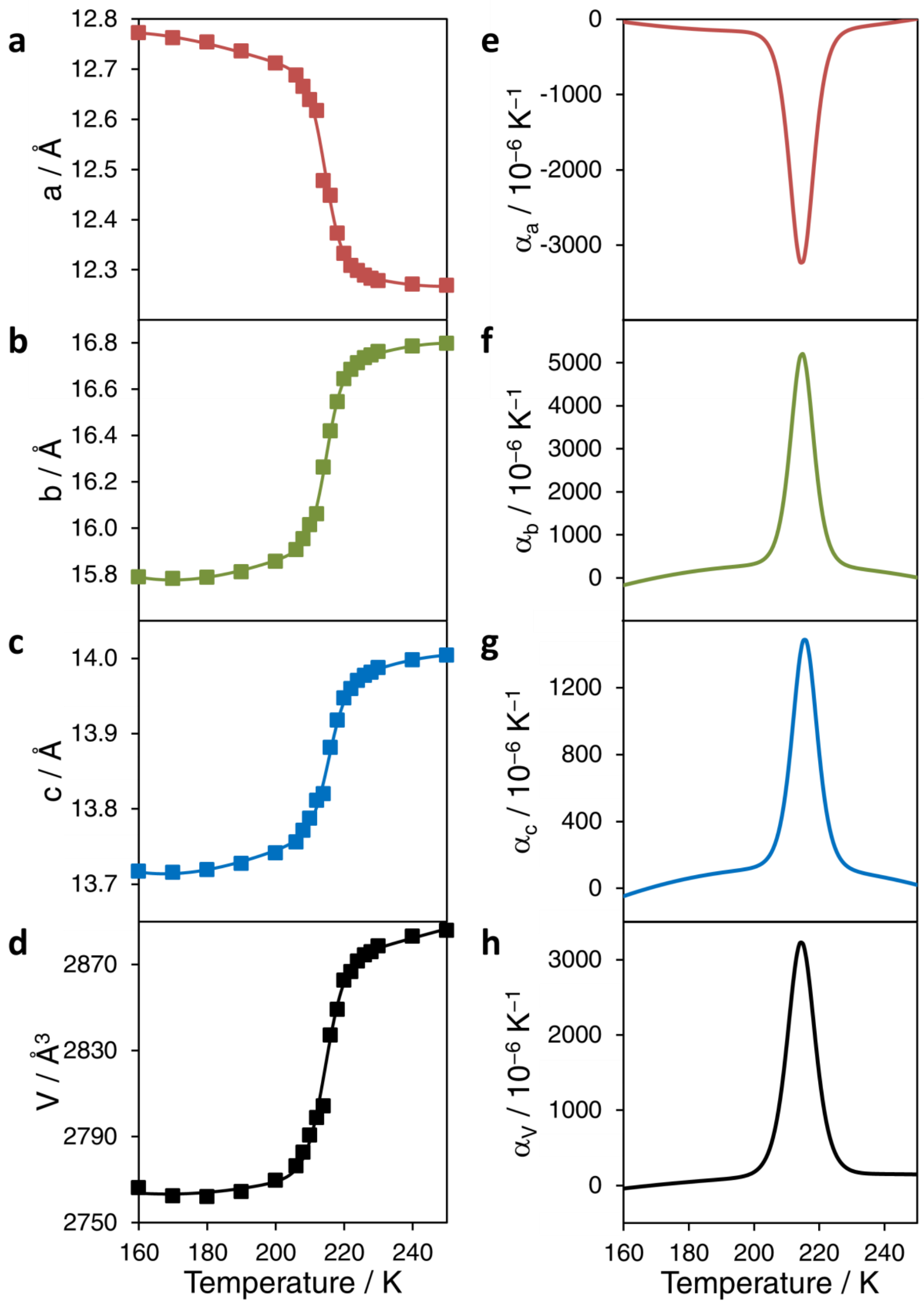

Supplementary Figure 9 | Unit cell dimensions (points) for x = 0.84 from 160 to 280 K, with supplementary equation (1) model fit (line): **a**, *a* parameter. **b**, *b* parameter. **c**, *c* parameter. **d**, volume. The corresponding coefficients of thermal expansion, α: **e**, *a* parameter. **f**, *b* parameter. **g**, *c* parameter. **h**, volume.

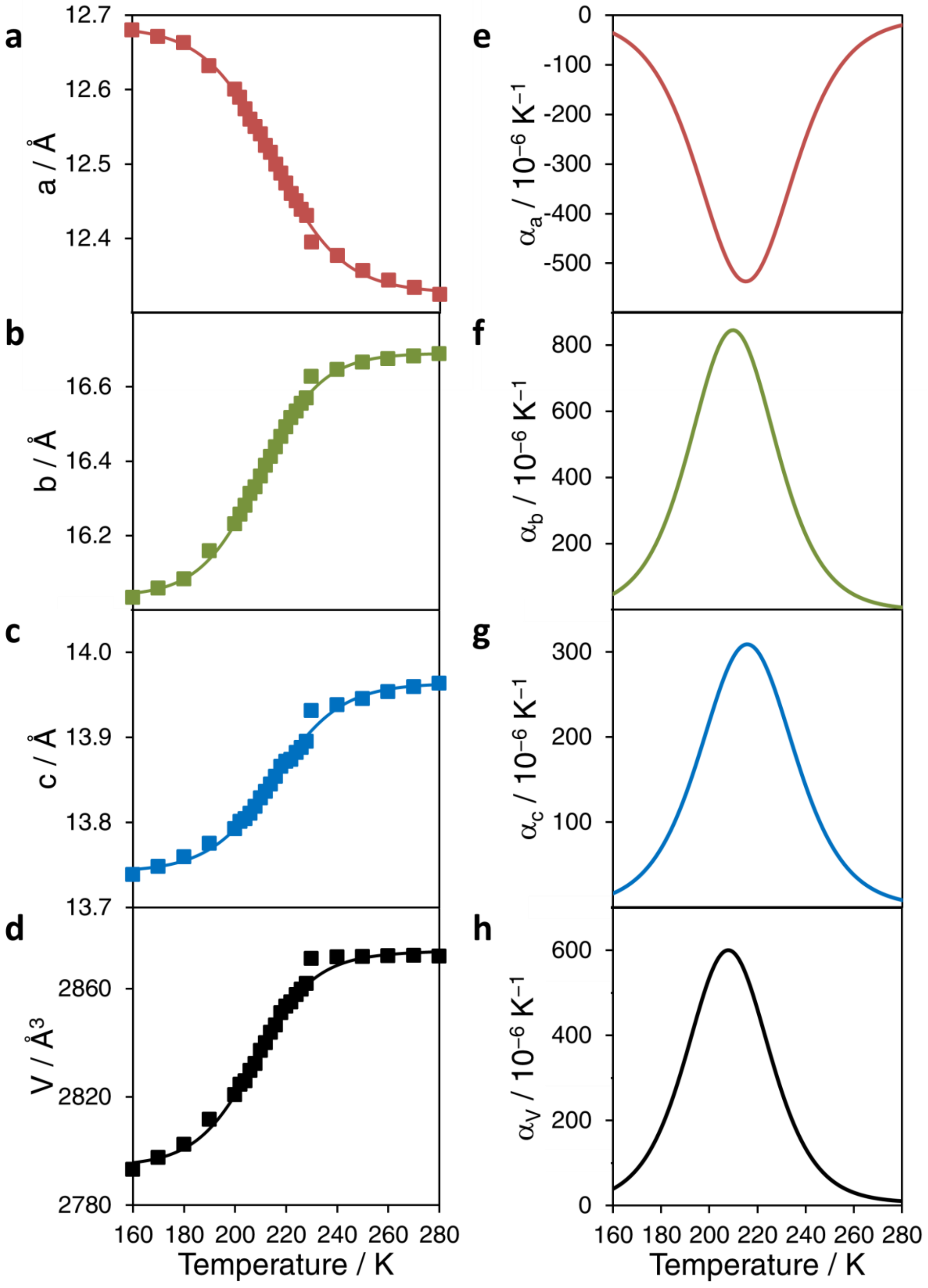

Supplementary Figure 10 | Unit cell dimensions (points) for x = 0.68 from 160 to 280 K, with supplementary equation (1) model fit (line): **a**, *a* parameter. **b**, *b* parameter. **c**, *c* parameter. **d**, volume. The corresponding coefficients of thermal expansion, α: **e**, *a* parameter. **f**, *b* parameter. **g**, *c* parameter. **h**, volume.

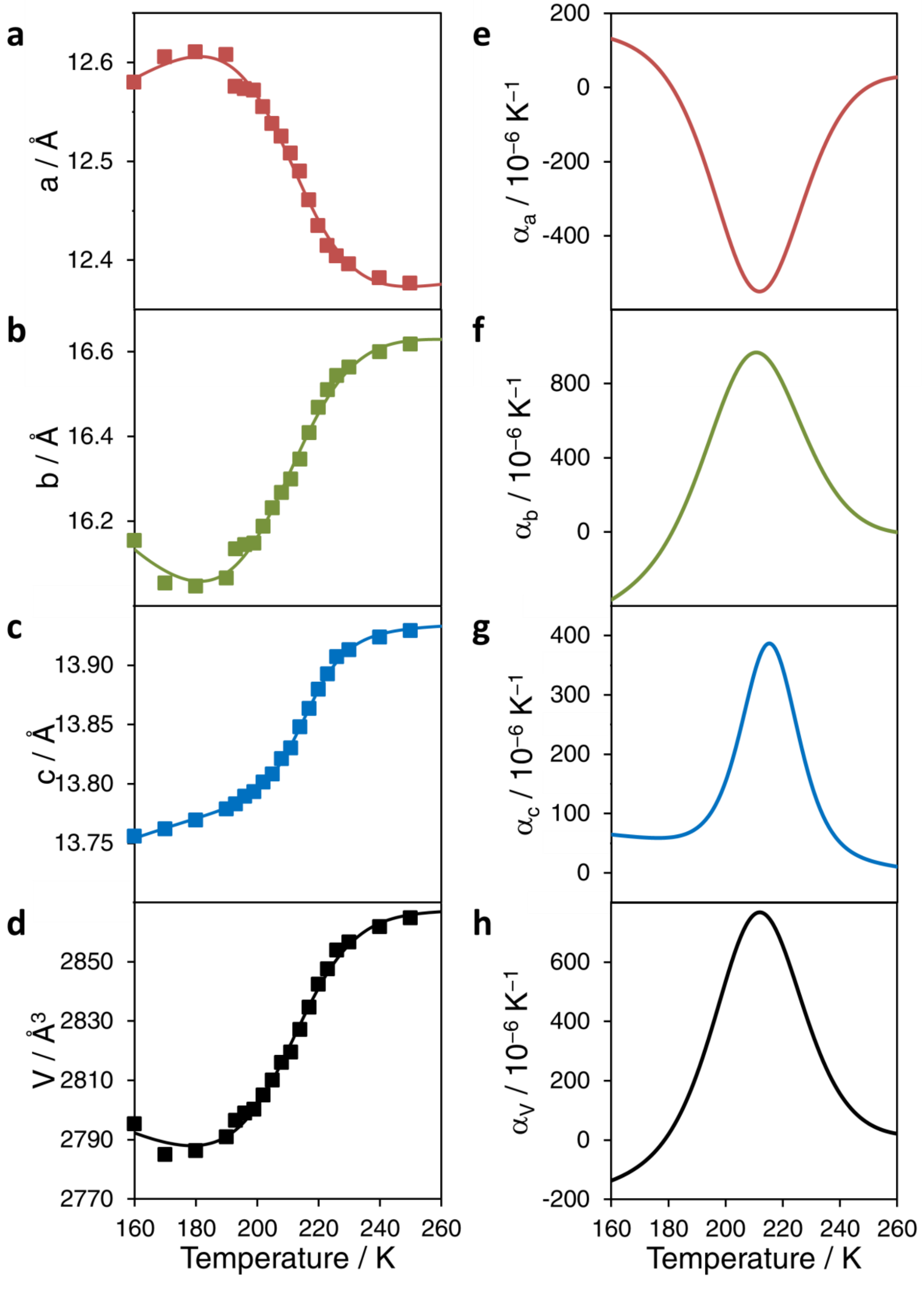

Supplementary Figure 11 | Unit cell dimensions (points) for *x* = 0.57 from 160 to 260 K with supplementary equation (1) model fit (line): **a**, *a* parameter. **b**, *b* parameter. **c**, *c* parameter. **d**, volume. The corresponding coefficients of thermal expansion, α: **e**, *a* parameter. **f**, *b* parameter. **g**, *c* parameter. **h**, volume.

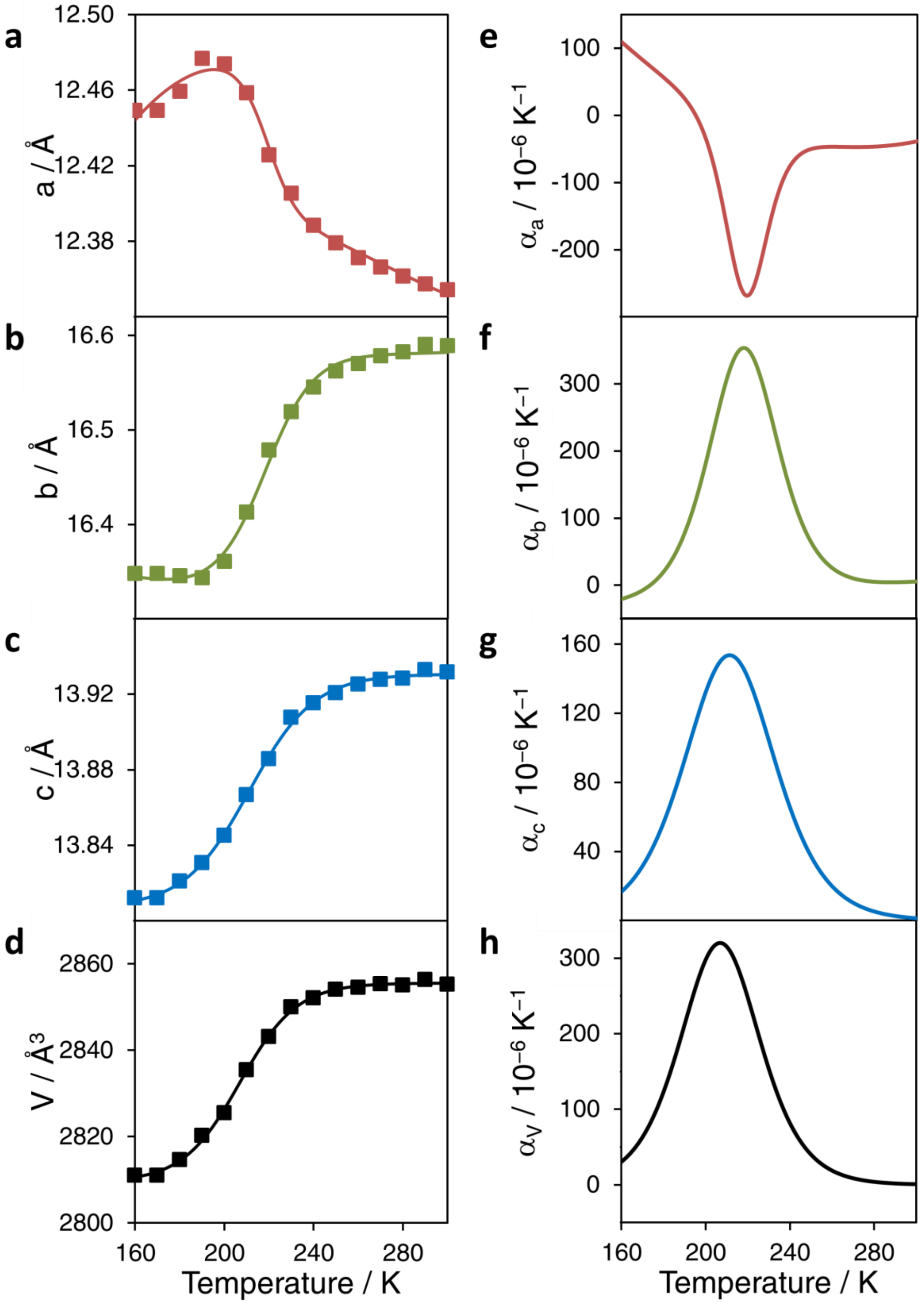

Supplementary Figure 12 | Unit cell dimensions (points) for x = 0.35 from 160 to 300 K with supplementary equation (1) model fit (line): **a**, *a* parameter. **b**, *b* parameter. **c**, *c* parameter. **d**, volume. The corresponding coefficients of thermal expansion, α: **e**, *a* parameter. **f**, *b* parameter. **g**, *c* parameter. **h**, volume.

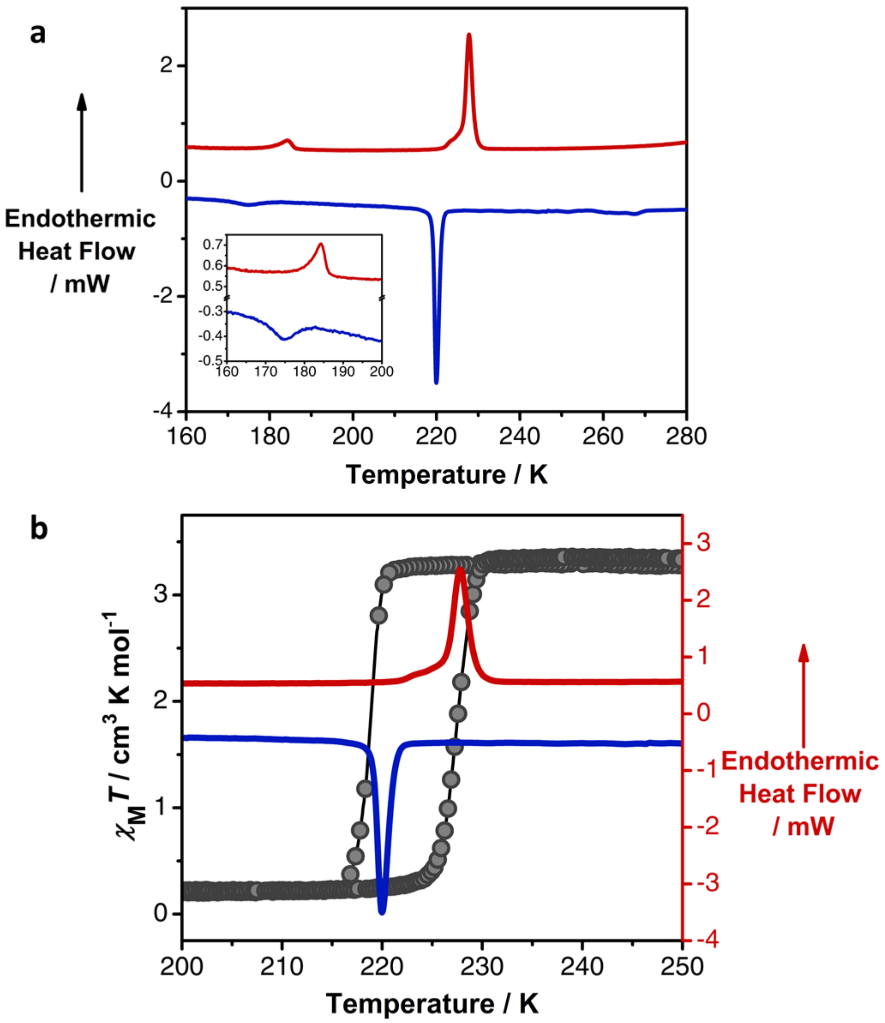

Supplementary Figure 13 | a, DSC scan of **[Fe]** between 160 and 280 K, upon cooling (blue line) and warming (red line). The inset highlights the heat flow response of the low temperature phase transition; **b**, comparison of the spin crossover as measured by magnetic susceptibility (grey spheres), and DSC upon cooling (blue) and warming (red).

Supplementary Table 1 | Comparison of iron fraction (x) values for the series **[Fe_xNi_{1-x}]** as calculated from the synthesis quantities, and found experimentally by electron microprobe analysis; and the relative atomic quantity of Au (expected: 2.0). These values are broadly consistent with the expected ratio within the uncertainty of the measurement, in which errors in the Fe/Ni composition are compounded in the calculation of [Au]. No crystalline and/or paramagnetic impurities are evident through the structural and magnetic studies, and it is noted that any minor amorphous impurities suggested by these elemental analysis data would not impact on the structural analyses.

***x* calculated**	***x* found**	**[Au] found**
**0.90**	0.84	1.70
**0.80**	0.68	1.89
**0.70**	0.57	1.99
**0.50**	0.35	1.97
**0.30**	0.25	2.20

Supplementary Table 2 | Summary of the single crystal X-ray diffraction collection and refinement data for $[Fe(bpac)(Au(CN)_2)_2]\cdot 2EtOH$ at 100, 190 and 240 K.

$[Fe(bpac)(Au(CN)_2)_2]\cdot 2EtOH$ at Temperature			
	100(2) K	190(2) K	240(2) K
CCDC deposition number	1534111	1501291	1501292
Formula	$C_{16}H_8Au_2FeN_6\cdot 2(C_2H_6O)$	$C_{16}H_8Au_2FeN_6\cdot 2(C_2H_6O)$	$C_{16}H_8Au_2FeN_6\cdot 2(C_2H_6O)$
Formula weight / g mol^{-1}	826.20	826.20	826.20
Crystal size / mm^3	0.13 × 0.13 × 0.06	0.13 × 0.13 × 0.06	0.13 × 0.13 × 0.06
Wavelength / Å	0.71073	0.71073	0.71073
Crystal system	Orthorhombic	Orthorhombic	Orthorhombic
Space group	*Cmma*	*Cmma*	*Cmma*
a / Å	12.1331(11)	12.5570(6)	11.707(2)
b / Å	16.0387(14)	15.8316(9)	17.442(4)
c / Å	13.6761(12)	13.6771(8)	14.117(3)
V / Å^3	2661.4(4)	2719.0(3)	2882.6(11)
Z	4	4	4
ρ_{calc} / Mg.m^{-3}	2.062	2.018	1.904
μ / mm^{-1}	11.560	11.315	10.673
F(000)	1528	1528	1528
Theta range / °	3.36 to 36.31	3.24 to 28.28	3.48 to 26.36
Index ranges	$-20 \le h \le 20$ $-26 \le k \le 24$ $-22 \le l \le 22$	$-16 \le h \le 13$ $-21 \le k \le 17$ $-10 \le l \le 18$	$-11 \le h \le 14$ $-18 \le k \le 21$ $-10 \le l \le 17$
Reflections collected	29883	8111	4537
Independent reflections [R_{int}]	3422	1821 [0.0350]	1553 [0.0610]
Completeness to θ / %	99.3% (to 36.31°)	99.2% (to 28.28°)	97.3% (to 25.00°)
Data/restraints/ parameters	3422 / 112 / 113	1821 / 17 / 102	1553 / 75 / 102
Goodness-of-fit on F^2	1.232	0.998	1.042
R indices, I > 2σ(I), (R_1,[(a)] wR_2[(b)])	0.0460, 0.0982	0.0289, 0.0780	0.0670, 0.1664
R indices, all data, (R_1,[(a)] wR_2[(b)])	0.0625, 0.1054	0.0372, 0.0859	0.1102, 0.2126
Largest peak and hole / e.Å^{-3}	5.28 and −4.10	2.85 and −1.39	5.40 and −1.88

(a) $R_1 = \Sigma \mid |F_o| - |F_c| \mid / \Sigma |F_o|$, (b) $wR_2 = \{\Sigma [w(F_o^2 - F_c^2)^2] / \Sigma [w(F_o^2)^2]\}^{1/2}$

Supplementary Table 3 | Parameters for the equation (1) model fits to the lattice parameters for $x = 0.84$. See Supplementary Fig. 9 for the corresponding plots of variable temperature lattice parameters and coefficients of thermal expansion.

Unit cell parameter	Equation (1) model parameters						
	A	***B***	***C***	***D***	***E***	***F***	***G***
a	-3.797×10^{-1}	214.4	2.451	10.65	3.462×10^{-2}	-1.816×10^{-4}	3.0×10^{-7}*
b	7.569×10^{-1}	214.8	2.355	24.17	-1.277×10^{-1}	6.307×10^{-4}	-1.0×10^{-6}*
c	1.939×10^{-1}	215.5	2.545	16.21	-3.828×10^{-2}	1.896×10^{-4}	-3.0×10^{-7}*
**Volume**	98.04	214.5	2.799	3075	−4.519	2.071×10^{-2}	-2.885×10^{-5}

*The model did not refine this parameter.

Supplementary Table 4 | Parameters for the equation (1) model fits to the lattice parameters for $x = 0.68$. See Supplementary Fig. 10 for the corresponding plots of variable temperature lattice parameters and coefficients of thermal expansion.

Unit cell parameter	Equation (1) model parameters						
	A	***B***	***C***	***D***	***E***	***F***	***G***
a	-3.500×10^{-1}	214.9	13.16	12.69	-5.908×10^{-3}	0	0
b	6.590×10^{-1}	210.0	11.88	16.04	-3.826×10^{-5}	0	0
c	2.218×10^{-1}	215.8	12.95	13.74	0	0	0
**Volume**	77.55	208.0	11.48	2794	-6.708×10^{-4}	3.327×10^{-5}	0

Supplementary Table 5 | Parameters for the equation (1) model fits to the lattice parameters for $x = 0.57$. See Supplementary Fig. 11 for the corresponding plots of variable temperature lattice parameters and coefficients of thermal expansion.

**Unit cell parameter**	**Equation (1) model parameters**						
	A	***B***	***C***	***D***	***E***	***F***	***G***
a	-3.651×10^{-1}	211.4	10.81	12.40	-4.616×10^{-4}	1.591×10^{-5}	$-3.5\times10^{-8*}$
b	9.008×10^{-1}	210.1	11.55	18.21	-2.001×10^{-2}	4.819×10^{-5}	$-3.0\times10^{-8*}$
c	1.283×10^{-1}	215.4	6.552	13.52	2.102×10^{-3}	$-3.8\times10^{-6*}$	0
**Volume**	100.1	211.7	10.43	2917	-1.117	2.090×10^{-3}	0

*The model did not refine this parameter

Supplementary Table 6 | Parameters for the equation (1) model fits to the lattice parameters for $x = 0.35$. See Supplementary Fig. 12 for the corresponding plots of variable temperature lattice parameters and coefficients of thermal expansion.

Unit cell parameter	Equation (1) model parameters						
	A	***B***	***C***	***D***	***E***	***F***	***G***
a	-8.606×10^{-1}	219.0	6.709	11.58	1.067×10^{-2}	-4.107×10^{-5}	5.0×10^{-8}
b	1.652×10^{-1}	218.1	10.96	16.46	-1.061×10^{-3}	1.9×10^{-6}*	0
c	1.235×10^{-1}	211.2	14.48	13.81	0	0	0
Volume	46.07	207.0	12.70	2810	0	0	0

*The model did not refine this parameter

Supplementary Table 7 | Comparison of spin crossover quantities with varying dilution proportions of **[Fe_xNi_{1-x}]**: maximum thermal expansion coefficients at the spin transition for the *a* ($\alpha_{a\ \mathrm{sco}}$), *b* ($\alpha_{b\ \mathrm{sco}}$), *c* ($\alpha_{c\ \mathrm{sco}}$) and volume ($\alpha_{V\ \mathrm{sco}}$) dimensions; the spin transition temperature as measured by powder X-ray diffraction, determined along the *c* axis (T_{XRD}); the spin transition temperature as measured by magnetic susceptibility (T_{mag}); and the width of the transition determined by the peak width at half maximum of the α_V plots.

x	**$\alpha_{a\ \mathrm{sco}}$ / 10^{-6} K^{-1}**	**$\alpha_{b\ \mathrm{sco}}$ / 10^{-6} K^{-1}**	**$\alpha_{c\ \mathrm{sco}}$ / 10^{-6} K^{-1}**	**$\alpha_{V\ \mathrm{sco}}$ / 10^{-6} K^{-1}**	**T_{XRD} / K**	**T_{mag} / K**	**SCO Width / K**
**0.84**	−3200	5200	1500	3200	216	221	10
**0.68**	−540	850	310	600	216	218	41
**0.57**	−550	970	390	770	215	216	37
**0.35**	−270	350	150	320	218	217	45

Supplementary Note 1. Magnetic behaviour of [Fe] and [Fe$_x$Ni$_{1-x}$]

[Fe(bpac)(Au(CN)$_2$)$_2$]·2EtOH (Fig. 2a): From room temperature to 230 K, the molar magnetic susceptibility product $\chi_M T$ is almost constant around 3.20 cm^3 K mol^{-1}, corresponding to Fe(II) in the high spin (HS) state with a slight anisotropy of g (2.06). As the temperature is lowered below 230 K a sharp decrease in $\chi_M T$ is observed to a value of 0.38 cm^3 K mol^{-1}, which is typical of a cooperative spin crossover to the low spin (LS) state with a transition temperature of $T_{1/2}^{\downarrow}$ = 221 K. Upon further cooling, $\chi_M T$ decreases slightly to 0.31 cm^3 K mol^{-1} at 100 K. On warming, χ_MT abruptly increases above 220 K as the material reverts back to the HS state, with $T_{1/2}^{\uparrow}$ = 226 K, producing a hysteresis width of approximately 5 K.

[Fe$_x$Ni$_{1-x}$(bpac)(Au(CN)$_2$)$_2$]·2EtOH (Fig. 3): The magnetic susceptibility response of the **[Fe$_x$Ni$_{1-x}$]** series arises from the contribution of both Fe(II) and Ni(II) metal centres, and the most suitable method for comparison is using the HS fraction of Fe(II), γ_{HS}.

The experimental molar magnetic susceptibility, $\chi_{exp}T$, can be described by the relationship $\chi_{exp}T = x \cdot \chi_{Fe}T + (1 - x) \cdot \chi_{Ni}T$ (χ_{Ni} = measured magnetic susceptibility in the pure Ni(II) complex; x = Fe(II) molar fraction). From this, the $\chi_{Fe}T$ contribution can be isolated, which is directly proportional to the HS fraction (γ_{HS}) of Fe(II) sites, since LS Fe(II) is diamagnetic.

Supplementary Note 2. Single crystal X-ray diffraction structures

At both temperatures the single crystal exists in the orthorhombic space group *Cmma*. Disordered guest ethanol molecules were crystallographically modelled inside the pores (2 EtOH per formula unit) of all structures.

The pyridyl rings of the bpac ligand in the three structures were modelled to be disordered over two orthogonal positions. The relative occupancy of each position was refined as a single free variable for each ring of the ligand, giving ratios for the A:B alignment of 67:33 (N(2) py) and 66:34 (N(3) py) at 240 K, 94:6 (N(2) py) and 95:5 (N(3) py) at 190 K, and 77:23 (N(2) py) and 74:26 (N(3) py) at 100 K; these values reflect the dynamic nature of the ligand when bound to HS Fe(II) at 240 K, with almost complete freezing to the A alignment achieved upon conversion to LS Fe(II) at 190 K. Interestingly, the ligand disorder becomes more pronounced as the framework structure relaxes back to a more compressed state at 100 K. Hydrogen atoms were modelled at calculated positions on the bpac ligand, but not on the ethanol guest molecule. The ethanol guest molecule was modelled as disordered over two positions, in which the oxygen atom is half occupied over two sites at either end of the C–C chain. Atom-to-atom distances within the ethanol molecule were restrained to appropriate values using the DFIX command. All non-hydrogen atoms were modelled anisotropically except for four carbon atoms (C(2B), C(3B), C(8B) and C(9B)) of the disordered pyridyl rings of the bpac ligand, and disordered ethanol atoms.

The *c* parameter is equivalent to the Fe···Fe distance across the bpac ligand, and the decrease in this quantity that accompanies the HS to LS transition is directly related to the decrease in the $Fe–N_{pyridyl}$ bond length. From 240 to 190 K the *c* parameter contracts by 0.439(4) Å, which corresponds well to the combined HS to LS contraction of the two $Fe–N_{pyridyl}$ bond lengths along this dimension, of 0.41(5) Å. The *c*-parameter remains almost constant (within error) to 100 K, which is consistent with the constant low spin state.

The more orthogonally octahedral coordination environment of the LS Fe(II) centre can be attributed to repulsive interactions between the ligand orbitals and the fully occupied t_{2g} metals orbitals, which lie between the ligand axes. The Fe(II) LS state also induces a more linear coordination of the cyanide ligands, due to the more efficient metal–ligand orbital overlap of

the σ bond, and to optimise π back-donation from the occupied metal orbitals to the antibonding cyanide orbitals.

It is worth noting the difference in thermomechanical behaviour observed by powder and single crystal X-ray diffraction experiments. These differences may be attributed to a matrix solvation effect: the PXRD measurement took place in a controlled solvent environment in a capillary whereas the crystal in the SCXRD experiment was coated in Paratone® oil and left open to the atmosphere. This material has demonstrated extreme flexibility which is affected by subtle differences in its environment, and would thus exhibit variable lattice flexing behaviour based on internal solvent effects in the sample. External strain associated with the rigid attachment of the crystal to the mounting fibre with frozen oil may also influence the lattice geometry of this highly flexible framework.

Supplementary Note 3. Synchrotron powder X-ray diffraction

A comparison of the diffraction data for the **[Fe_xNi_{1-x}]** series at 250 K is shown in Fig. S4. All powder patterns were able to be fully indexed with the orthorhombic *Cmma* unit cell and were consistent with the pattern simulated from the single crystal X-ray diffraction data of **[Fe]**, indicating the absence of any polycrystalline impurities. The close similarity of all patterns confirms that the series is isostructural, with the small, continuous variations in peak position observed upon Ni(II) doping resulting in part from the intermediate size of Ni(II) (ionic radius 83 pm), compared to HS Fe(II) (92 pm) and LS Fe(II) (75 pm).

The variable temperature diffraction data for the **[Fe_xNi_{1-x}]** materials display smooth, continuous lattice changes over the spin transition (see Supplementary Fig. 5 for representative data). The shifts in peak positions with temperature greatly exceed the small degree of peak broadening observed over the spin transition, confirming sample homogeneity. Moreover, the peak broadening over the transition is comparable to that observed for non-doped, gradual spin crossover materials, and can therefore be attributed principally to strain broadening associated with a distribution of HS and LS Fe(II) sites within the crystal lattice, rather than to sample broadening (which may, for example, arise from variations in the value of *x* and/or varying size of individual crystallites, leading to a distribution of spin crossover temperatures).

Unit cell parameters were modelled by Le Bail refinement within *GSAS*[9] using the *EXPGUI*[10] interface (see Supplementary Fig. 6 for a representative example). A histogram profile function with a pseudo-Voigt peak shape and a 10 to 16 term shifted Chebyshev background function were used. Peak profile and unit cell parameters were refined. Sequential refinements were performed using a software script, in which the starting parameters for a refinement were taken from the parameters of the previous temperature in the series.

Supplementary Note 4. Lattice parameter model of [Fe_xNi_{1-x}]

The variable temperature lattice parameter data for **[Fe_xNi_{1-x}]** were modelled using equation (1), denoted *L(T)*, which combines a sigmoidal function with a 3rd order polynomial:

$$L(T) = \frac{A}{\left(1 + \exp\left(\frac{(B-T)}{C}\right)\right)} + D + ET + FT^2 + GT^3 \qquad (1)$$

The sigmoidal function was incorporated as it was found empirically to provide an excellent fit in the region of the spin crossover transition. This function has been previously used to fit structural changes in a spin crossover material[11]. The equation parameters represent: ***A***, the change in the cell parameter over the spin transition (negative for NTE, positive for PTE); ***B***, the sigmoidal centre, approximately equivalent to the magnetic spin transition temperature, T_{sco}; ***C***, the sigmoidal width, related to the abruptness of the spin crossover transition; and the polynomial parameters ***D***, ***E***, ***F*** and ***G*** are required to fit the lattice parameter behaviour outside of the spin transition temperature range. The same function was used to separately fit the volume data. The resulting fitting parameters for **[Fe_xNi_{1-x}]** (x = 0.84, 0.68, 0.57 and 0.35), are given in Supplementary Tables 3, 4, 5 and 6, respectively.

The coefficients of linear and volumetric thermal expansion are proportional to the first derivative of the unit cell model formula with respect to temperature, and were calculated according to equation (2):

$$\alpha(T) = \frac{1}{L(T)} \cdot \frac{\partial L(T)}{\partial T} = \frac{1}{L(T)} \left\{ \frac{A \cdot \exp\left(\frac{(B-T)}{C}\right)}{C \cdot \left(1 + \exp\left(\frac{(B-T)}{C}\right)\right)^2} + E + 2FT + 3GT^2 \right\} \qquad (2)$$

Supplementary Note 5. Coefficients of Thermal Expansion

$[Fe(bpac)(Au(CN)_2)_2]\cdot 2EtOH$

As shown in Supplementary Fig. 7, the lattice parameters of **[Fe]** display remarkable variation over the temperature range studied. In addition to the extreme degree of lattice flexing over the spin transition, there is additional continuous lattice movement at lower temperature. Between 210 K and 100 K the coefficients of linear thermal expansion range between 85×10^{-6} K^{-1} > α_a > -1100×10^{-6} K^{-1} along the *a*-axis, and 860×10^{-6} K^{-1} > α_b > -39×10^{-6} K^{-1} along the *b*-axis (Supplementary Fig. 7). This behaviour likely arises due to a temperature dependence on the distortive influences on the framework geometry, such that these influences become weaker as the temperature is decreased. Such influences could include inter-lattice and lattice–guest interactions, which would weaken as atomic vibrations undergo thermal contraction. Eventually the energetics reach a critical point, where the distortive force is too weak to counteract the geometrical strain of the more rigidly octahedral LS Fe(II), and the material undergoes the second structural transition at 170 K.

$[Fe_xNi_{1\text{-}x}(bpac)(Au(CN)_2)_2]\cdot 2EtOH$

Supplementary Figs. 9–12 display the temperature-dependence on the unit cell parameters of the Ni(II)-doped materials, **[Fe_xNi_{1-x}]**, refined from powder X-ray diffraction; the model fit to these data; and the corresponding coefficients of thermal expansion.

Supplementary Note 6. Differential Scanning Calorimetry

The energy changes associated with the phase transitions of the pure **[Fe]** material were studied using differential scanning calorimetry (DSC).

The calorimetric measurements were performed using a Mettler Toledo DSC823e instrument. A sample of **[Fe]** (2.71 mg) was hermetically sealed in an aluminium pan under a thin layer of ethanol solvent. Heating and cooling cycles were run under a constant flow of dry dinitrogen gas (0.5 L·min^{-1}) and a sealed empty aluminium pan was used as a reference. Data were acquired in the range 150–298 K at a thermal ramp rate of 5 K·min^{-1}. After the run cycle had finished, the aluminium sample pan was punctured, and the sample was dried under dry dinitrogen gas flow (0.5 L·min^{-1}) at 45 °C for 1.5 h to allow determination of the sample weight. DSC scan peaks were integrated to determine enthalpic and entropic values for the phase transitions.

The DSC results clearly show the spin transitions upon cooling and heating (Supplementary Fig. 13). Both transitions align well with those observed in the magnetic data (Supplementary Fig. 13b), with an exothermic transition at 220 K upon cooling (ΔH = −14 kJ·mol^{-1}, ΔS = −64 J·K^{-1}·mol^{-1}) and an endothermic transition at 228 K upon warming (ΔH = 15 kJ·mol^{-1}, ΔS = 67 J·K^{-1}·mol^{-1}). These enthalpic and entropic values for the spin transition are comparable to those of previously reported framework systems[12].

Corroborating the results observed in the powder X-ray diffraction experiments, the DSC scan (Supplementary Fig. 13a) also displays the low-temperature phase transition as an exothermic process upon cooling centred at 174 K (ΔH = −1.6 kJ·mol^{-1}, ΔS = −9.4 J·K^{-1}·mol^{-1}), and an endothermic transition upon heating centred at 184 K (ΔH = 1.9 kJ·mol^{-1}, ΔS = 11 J·K^{-1}·mol^{-1}).

Supplementary Methods

Synthesis of ligand 1,2-bis(4′-pyridyl)acetylene (bpac).

1,2-Bis(4′-pyridyl)acetylene (bpac) was synthesized from 1,2-dibromo-1,2-bis(4′-pyridyl)ethane using published methods[1] and confirmed using ^{1}H NMR in *d*-chloroform.

1,2-Dibromo-1,2-bis(4′-pyridyl)ethane: *Trans*-1,2-bis(4′-pyridyl)ethylene (2.03 g, 11.0 mmol) was dissolved in concentrated hydrobromic acid (48%, 20 mL) at 0 °C. Bromine (7.13 g, 44.6 mmol) was added to the suspension with stirring. The reaction mixture was heated at reflux for 1 h, then cooled to 0 °C. The precipitate was isolated by filtration, then treated with NaOH (2 M, 4 × 20 mL), washed with water (3 × 15 mL) and dried. The solid was then dissolved in a mixture of dichloromethane and chloroform (3:2, 200 mL), dried over anhydrous sodium sulfate, and filtered. The solvent was removed under reduced pressure to give 1,2-dibromo-1,2-bis(4′-pyridyl)ethane (3.66 g, 10.7 mmol, 97%) as a fine white powder. ^{1}H NMR (300 MHz, $CDCl_3$): δ 5.28 (2H, s, C*H*Br), 7.40 (4H, d, *J* = 4.5 Hz, Pyr*H*), 8.69 (4H, d, *J* = 4.5 Hz, Pyr*H*). ^{13}C NMR (300 MHz, $CDCl_3$): δ 150.47, 147.61, 122.56, 51.78.

1,2-Bis(4′-pyridyl)acetylene (bpac): 1,2-dibromo-1,2-bis(4′-pyridyl)ethane (2.12 g, 6.20 mmol) was dissolved in *tert*-butanol (40 mL) and added dropwise to a solution of potassium *tert*-butoxide (2.8 g, 28 mmol) in *tert*-butanol (50 mL) at 110 °C. The reaction mixture was heated at reflux for 1 h. Water (3 mL) was added and the solvent removed under reduced pressure. The crude product was extracted into diethyl ether (3 × 20 mL), dried over anhydrous sodium sulfate and filtered. The ether extract was evaporated to dryness and the resulting solid was recrystallized from hexane to give 1,2-bis(4′-pyridyl)acetylene (0.559 g, 3.07 mmol, 50%) as pale yellow crystals. ^{1}H NMR (300 MHz, $CDCl_3$): δ 7.41 (4H, d, *J* = 4.5 Hz, Pyr*H*), 8.65 (4H, d, *J* = 4.5 Hz, Pyr*H*). ^{13}C NMR (300 MHz, $CDCl_3$): δ 150.34, 130.63, 125.99, 91.01. mp = 115–117 °C.

Synthesis of $[Fe_xNi_{1-x}(bpac)(Au(CN)_2)_2]\cdot 2EtOH$ ($0 \leq x \leq 1$).

$[Fe(bpac)(Au(CN)_2)_2]\cdot 2EtOH$, ***[Fe]***: Iron perchlorate hydrate, $Fe(ClO_4)_2\cdot 9H_2O$ (0.14 mmol, 58.2 mg; *Caution:* perchlorate salts are potentially explosive and should be handled with care; only small quantities should be prepared) was dissolved in ethanol (50 mL) and slowly added to a solution

of 1,2-bis(4′-pyridyl)acetylene, bpac (0.14 mmol, 25.2 mg) and potassium dicyanidoaurate, $KAu(CN)_2$ (0.28 mmol, 80.7 mg) in ethanol (50 mL). The compound rapidly precipitated as a yellow powder, which was stirred for 1 h then centrifuged, washed with water (20 mL) to remove potassium perchlorate impurity, then with ethanol (2 × 20 mL). The sample was stored under ethanol solvent and all structural and magnetic measurements were performed with care taken to prevent ethanol desorption prior to or during the measurement. For elemental microanalyses, samples were desorbed with heating to 100 °C immediately prior to measurement. *Anal.* Found: C, 25.8; H, 1.2; N, 11.1; Fe, 7.6; Au, 53.9%. Calc. for $C_{16}H_8N_6FeAu_2$: C, 26.2; H, 1.1; N, 11.4; Fe, 7.6; Au, 53.7%.

[Fe_xNi_{1-x}(bpac)($Au(CN)_2)_2$]·2EtOH: Members of the series **[Fe_xNi_{1-x}]** ($0 < x < 1$) were prepared according to the above procedure, with $Fe(ClO_4)$·$9H_2O$ and $Ni(ClO_4)_2$·$6H_2O$ dissolved in ethanol in $x : (1 - x)$ molar ratios, such that $x = 0.30$, 0.50, 0.70, 0.80 and 0.90. The Ni dilution quantity was analysed by energy dispersive X-ray spectroscopy, and thus reliable relative quantities were only obtained for the heavy elements Fe, Ni and Au. Relative atomic values for these elements were calculated such that [Fe] + [Ni] = 1.0 (Supplementary Table 1, Supplementary Fig. 1).

Magnetic Susceptibility Measurements

Magnetic properties were measured on a MPMS-XL7 Quantum Design SQUID magnetometer. After measurement at 1 Tesla of applied magnetic field under ethanol solvent, the sample was dried and weighed to determine $\chi_M T$ values.

Single Crystal X-ray Diffraction Experiments

A summary of collection and refinement details is contained in Supplementary Table 2. Single crystals of [Fe(bpac)($Au(CN)_2)_2$]·2EtOH, **[Fe]**, were grown by slow diffusion of a 1:2:1 molar ratio of $Fe(ClO_4)_2$·$9H_2O$, K[$Au(CN)_2$] and bpac in ethanol. An appropriate single crystal was selected under a polarising microscope and mounted in a Kapton capillary in a thin film of grease. Data were collected on a Bruker-Nonius FR591 Kappa APEX II equipped with Mo-Kα (0.71073 Å) and an Oxford Instruments nitrogen gas cryostream. The crystal was first quench-cooled in the cryostream at 100 K, then data collections were performed at 100, 190 and 240 K, below the low temperature phase transition, and below and above the spin crossover transition, respectively.

Data collection, cell refinement and data reduction were performed within the *APEX2* (v. 2009.3-0) [2] software suite. The structures were solved with *SHELXS-97*[3] using direct methods and refined using *SHELXL-97*[4] and *SHELXL-2013*[5], through the *X-Seed!* GUI[6]. Refinement details are given in Supplementary Table 2, and the asymmetric units of the two structures are shown in Supplementary Fig. 2.

Synchrotron Powder X-ray Diffraction Experiments

Powder X-ray diffraction was chosen to study the structural behaviour of **[Fe]** due to the superiority of this technique in determining variable temperature trends in unit cell parameter evolution over that of single crystal diffraction methods. Additionally, this technique provides analysis of the bulk material, allowing comparison with the bulk magnetic data.

Variable temperature powder X-ray diffraction experiments were conducted on the Powder Diffraction beamline at the Australian Synchrotron, using a wavelength of $\lambda = 0.60470$ Å.

Supplementary References

1. Tanner, M.L. & Ludi, A. A facile synthesis of 4,4'-dipyridylacetylene. *Chimia* **34**, 23–24 (1980)
2. Bruker, *APEX2* and *SAINT* (Bruker AXS Inc., Madison, Wisconsin, USA, 2009).
3. Sheldrick, G. M. *SHELXS-97* (University of Göttingen, Göttingen, Germany, 1997).
4. Sheldrick, G. M. *SHELXTL-97* (University of Göttingen, Göttingen, Germany, 1997).
5. Sheldrick, G. M. A short history of SHELX. *Acta Cryst. A* **A64**, 112–122 (2008).
6. Barbour, L. J. X-Seed – a software tool for supramolecular crystallography *Supramol. Chem.* **1**, 189–191 (2001).
7. van der Sluis, P. & Spek, A. L. BYPASS: an effective method for the refinement of crystal structures containing disordered solvent regions. *Acta Cryst.* **A46**(3), 194–201 (1990).
8. Richard, D., Ferrand, M. & Kearley, G.J. Analysis and visualisation of neutron-scattering data. *J. Neutron Res.* **4**, 33–39 (1996).
9. Larson, A.C. & Von Dreele, R.B. *General Structure Analysis System (GSAS)* (Los Alamos National Laboratory Report LAUR 86-748, 2000).
10. Toby, B. H. EXPGUI, a graphical user interface for GSAS *J. Appl. Crystallogr.* **34**, 210 (2001).
11. van der Veen, R. M., Kwon, O.-H., Tissot, A., Hauser, A. & Zewail, A. H. Single-nanoparticle phase transitions visualised by four-dimensional electron microscopy. *Nature Chem.* **5**, 395–402 (2013).
12. Niel, V., Martinez-Agudo, J. M., Muñoz, M. C., Gaspar, A. B., Real, J. A. Cooperative spin crossover behaviour in cyanide-bridged Fe(II)–M(II) bimetallic 3D Hofmann-like networks (M = Ni, Pd, Pt). *Inorg. Chem.* **40**, 3838–3839 (2001).
