# Supplementary material for: Spin crossover-induced colossal positive and negative thermal expansion in a nanoporous coordination framework material
Source: Nat Commun. 2017 Oct 20;8:1053. doi: 10.1038/s41467-017-00776-1 (PMC5648752; doi:10.1038/s41467-017-00776-1)
Supplement: Supplementary file 2 — Description of Additional Supplementary Files [file 41467_2017_776_MOESM2_ESM.pdf]

Description of Additional Supplementary Files

File Name: Supplementary Movie 1
Description: Animation showing the spin crossover-induced lattice flexing behaviour of the doped framework material, [Fe_{0.84}Ni_{0.16}].
